# From bugs to bots: learning infectious diseases in the AI era

**DOI:** 10.1017/ash.2025.10257

**Published:** 2025-12-17

**Authors:** Guillermo Rodriguez-Nava

**Affiliations:** 1 Department of Medicine, Denver Health and Hospital Authority, Denver, CO, USA; 2 Division of Infectious Diseases, University of Colorado School of Medicine, Aurora, CO, USA

## Dear Editor,

As a millennial, I have experienced the shift from an analog world to a digital universe. This transition has shaped many parts of my life, including my education. My journey in education began with government-issued textbooks during elementary school.^1^ It continued through the explosion of the internet and the World Wide Web, which fueled the rapid evolution of digital reference tools, such as encyclopedia Encarta and later Wikipedia. As digital connections grew faster, new tools emerged in the palm of my hand through smartphones; from digital books to podcasts and YouTube videos. However, as these learning resources become increasingly accessible, the sheer volume of information often exceeds what one can reasonably review or study. Furthermore, the more specialized a medical field becomes, the more clinicians must rely on lengthy guidelines, reviews, and research papers, many of which are updated far more rapidly than other sources. Now, a new tool has captured my attention and addresses this challenge. It even helped me prepare more efficiently for my Infectious Diseases board examination: large language models (LLMs).

LLMs deployed as conversational chatbot tools, such as ChatGPT, have captivated the world, including the medical community. They have demonstrated strong performance on standardized tests, the ability to generate humanlike responses, and empathetic communication.^2^ This has led to the anthropomorphization of chatbot tools, overshadowing tasks that LLMs truly excel at, such as data summarization, in the push to replicate human clinical reasoning. In fact, up to 82% of recent studies on LLMs in medicine have focused on question-answering, whereas only about 9% have examined summarization tasks.^3^

Spaced repetition is a learning method that improves long-term retention by reviewing material at intervals set by a spacing algorithm.^4^ This is a technique I began using effectively while preparing for the United States Medical Licensing Examination, and its effectiveness is well documented.^5^ When I began preparing for the Infectious Diseases boards, I struggled to decide what to study. The George Washington University Infectious Disease Board Review Course™ gave me a framework; however, when I needed to review primary sources, particularly guidelines, it was not practical to study fifty-page documents effectively. Then it clicked for me: I could use a chatbot LLM, specifically ChatGPT, to create flashcards for my spaced repetition software. Creating the flashcards was as simple as copying and pasting guideline sections or topics into ChatGPT and then personalizing the cards as needed. Table 1 provides a sample of these flashcards, illustrating how ChatGPT can convert guideline content into effective study tools.^6^

**Table 1.** Case-based flashcards summarizing key concepts on carbapenem-resistant *enterobacterales* from the IDSA 2024 Guidance on the treatment of antimicrobial resistant gram-negative infections

Card	Front (Case + Question)	Back (Answer / Key Points)
1	A 71-year-old woman with diabetes presents with dysuria and positive urine culture showing carbapenem-resistant *Enterobacterales*. What are preferred antibiotics for **uncomplicated cystitis** caused by CRE?	– Preferred options **if susceptible**: nitrofurantoin, TMP-SMX, ciprofloxacin, levofloxacin. – Alternatives: **single-dose aminoglycoside**, oral fosfomycin (E. coli only), colistin, ceftazidime-avibactam, meropenem-vaborbactam, imipenem-cilastatin-relebactam, cefiderocol. – Susceptibility to preferred oral agents is often **low**, but they achieve high urine concentrations.
2	A 66-year-old man with obstructive uropathy is hospitalized with fever and flank pain. Urine culture grows CRE *Klebsiella*. What are preferred antibiotics for **pyelonephritis or cUTI** caused by CRE?	– Preferred **if susceptible**: TMP-SMX, ciprofloxacin, levofloxacin. – Additional preferred: ceftazidime-avibactam, meropenem-vaborbactam, imipenem-cilastatin-relebactam, cefiderocol. – Aminoglycosides: **alternative** agents. – Fosfomycin **not recommended** for pyelonephritis/cUTI due to poor renal parenchymal penetration.
3	A 59-year-old man with CRE bacteremia has an isolate that is **not carbapenemase-producing** and is susceptible to meropenem/imipenem but resistant to ertapenem. What is the preferred treatment approach?	– For isolates **meropenem- or imipenem-susceptible but ertapenem-resistant**: use **extended-infusion meropenem** (or imipenem-cilastatin). – If **not susceptible to any carbapenem**: preferred agents are ceftazidime-avibactam, meropenem-vaborbactam, or imipenem-cilastatin-relebactam. – Avoid polymyxin- or aminoglycoside-based combinations due to **higher mortality and nephrotoxicity**.
4	A 72-year-old woman with CRE intra-abdominal infection has an isolate producing **KPC**. What are preferred antibiotics for infections caused by **KPC-producing CRE**?	– Preferred: **meropenem-vaborbactam**, **ceftazidime-avibactam**, **imipenem-cilastatin-relebactam**. – Meropenem-vaborbactam is **slightly preferred** (highest cure, lowest resistance emergence). – Cefiderocol: **alternative** agent (reserve for MBL producers or difficult cases). – Older regimens (polymyxins/aminoglycosides): worse outcomes and higher toxicity.
5	A 68-year-old man with CRE bloodstream infection tests positive for **NDM**. What are preferred treatments for infections caused by **MBL-producing CRE (e.g., NDM, VIM, IMP)**?	– Preferred: **ceftazidime-avibactam + aztreonam** (covers both serine β-lactamases and MBL). – Preferred alternative: **cefiderocol** monotherapy (≈90% active against MBL producers). – Avoid routine use of other β-lactams (none inhibit MBL). – Tigecycline/eravacycline: **alternative** only for non-urinary, non-bloodstream infections.

**Abbreviations:** AUC, area under the curve; BID, twice daily; CRE, carbapenem-resistant Enterobacterales; cUTI, complicated urinary tract infection; FDA, Food and Drug Administration; IMP, imipenem-hydrolyzing metallo-β-lactamase; IV, intravenous; KPC, *Klebsiella pneumoniae* carbapenemase; MBL, metallo-β-lactamase; MIC, minimum inhibitory concentration; NDM, New Delhi metallo-β-lactamase; PO, by mouth; TMP-SMX, trimethoprim-sulfamethoxazole; UTI, urinary tract infection; VIM, Verona integron-encoded metallo-β-lactamase.

Up until a month before the examination, I had built a deck of roughly 2,000 flashcards. This approach, combined with reviewing source material to ensure accuracy and using other study methods, made the process far more enjoyable. Of course, a single experience is unlikely to convince an evidence-based community of this strategy’s efficacy. However, my experience may encourage a formal evaluation of this approach or inspire future fellows to explore new strategies for board preparation. It may also help new infection preventionists learn the complex National Healthcare Safety Network healthcare-associated infection definitions, or support junior pharmacists in mastering antiretroviral drug interactions. One thing is certain: LLMs represent the next step in technological development. Those who do not learn to use them risk falling behind compared to those who do.
